# Evaluation of complications and biochemical recurrence rates after (super) extended lymph node dissection during radical prostatectomy

**DOI:** 10.1007/s00345-024-05321-6

**Published:** 2024-10-30

**Authors:** Diederik J.H. Baas, Bas Israël, Joost M.S. de Baaij, Henricus J.E.J. Vrijhof, Robert J. Hoekstra, Heidi Kusters-Vandevelde, Peter F.A. Mulders, J. P. Michiel Sedelaar, Diederik M. Somford, Jean-Paul A. van Basten

**Affiliations:** 1 Department of Urology, Canisius Wilhelmina Hospital, Weg door Jonkerbos 100, Nijmegen, 6532 SZ The Netherlands; 2 Department of Urology, Radboud University Medical Center, Nijmegen, The Netherlands; 3 Prosper Prostate Cancer Clinics, Nijmegen/Eindhoven, The Netherlands; 4 Department of Urology, Catharina Hospital, Eindhoven, The Netherlands; 5 Department of Pathology, Canisius Wilhelmina Hospital, Nijmegen, The Netherlands

**Keywords:** Prostate cancer, Prostatectomy, Pelvic lymph node dissection (PLND), Complications, Oncological outcomes, Biochemical recurrence

## Abstract

**Objective:**

To evaluate the effectiveness of extended (e-PLND) and super-extended pelvic lymph node dissection (se-PLND) during robot-assisted radical prostatectomy (RARP) by examining lymph node (LN) yield, complications, LN metastasis, and biochemical recurrence (BCR) incidence.

**Methods:**

Between January 2016 and January 2020, 354 consecutive patients with > 5% risk of lymph node involvement (LNI), as predicted by the Memorial Sloan Kettering Cancer Center nomogram, underwent RARP with (s)e-PLND at a high-volume center. The e-PLND involved removing fibrofatty lymphatic tissue around the obturator fossa, internal iliac region, and external iliac vessels. The se-PLND, performed at the discretion of the surgeons, also included lymph nodes from the pre-sacral and common iliac regions. Outcomes included histopathological findings by anatomical region; complications; and BCR incidence during follow-up.

**Results:**

The median LNI risk was 18% (IQR 9–31%). A median of 22 LN (IQR 16–28) were removed, with se-PLND yielding a higher number: 25 (IQR 20–32) compared to e-PLND: 17 (IQR 13–24) (*p* < 0.001). pN1 disease was detected in 22% of patients overall, higher in se-PLND (29%) than e-PLND (14%) (*p* < 0.001). Of metastatic LNs, 14% were situated outside the e-PLND template. Operation time was longer for se-PLND, but perioperative complications were similar between both groups. After a median follow-up of 24 months (IQR 7–33), BCR incidence was comparable between the two groups.

**Conclusion:**

Compared to standard extended pelvic lymph node dissection (PLND), super extended PLND increases lymph node yield and removal of metastatic deposits but does not contribute to progression free survival at mid-term.

**Supplementary Information:**

The online version contains supplementary material available at 10.1007/s00345-024-05321-6.

## Introduction

Treatment options and the prognosis for men with prostate cancer (PCa) are closely intertwined with the presence of metastasis, primarily situated in the pelvic lymph nodes (LN) [1]. Because both conventional imaging techniques and Prostate-Specific Membrane Antigen (PSMA) positron emission tomography (PET)/CT lack adequate sensitivity for direct detection of positive LNsc [2–7], current guidelines recommend the use of nomograms to estimate the risk of positive LNs. The European Association of Urology (EAU) PCa guidelines recommend the use of the web-based Memorial Sloan Kettering Cancer (MSKCC) and Briganti nomograms, Partin tables, and the Roach formula [8–11], with the MSKCC and Briganti nomogram being the most accurate [12]. These nomograms combine clinical tumor stage, initial serum prostate-specific antigen (PSA) levels, tumor aggressiveness determined by the International Society of Urological Pathology (ISUP) Grade Group (or Gleason Score [GS]), and the percentage of positive cores identified in prostate biopsies for the prediction of lymph node invasion (LNI) risk [8–10, 13].

According to these nomograms, the EAU guideline recommends an extended pelvic lymph node dissection (e-PLND) in addition to radical prostatectomy (RP) if the predicted LNI risk exceeds 5% for systematic biopsies and 7% for targeted biopsies [11]. An e-PLND includes dissection of the lymph nodes within the obturator fossa, overlying the external iliac artery and vein and internal iliac artery [14]. In cases with a high risk of LNI, a super extended PLND (se-PLND), which may include dissection of nodes in the presacral area and those overlying the common iliac artery, can be performed [15].

While a more extended PLND results in a higher number of removed lymph nodes and an increased detection rate of nodal metastasis, it is associated with adverse perioperative outcomes and longer operation times, potentially impacting the patient’s quality of life [14, 16]. However, e-PLND remains the established gold standard for assessing nodal status as any therapeutic benefit has not been demonstrated yet [14, 17, 18]. The pathological outcome of PLND is of importance to tailor adjuvant and/or salvage therapy and the EAU guidelines [11] state the following management options for pN1 disease considering nodal involvement characteristics: offer adjuvant androgen depressant therapy (ADT); offer adjuvant ADT with additional radiotherapy; offer observation. In the Netherlands, there is general consensus on observation as the most appropriate management option and in our series ADT was never prescribed before biochemical recurrence occurred, offering a unique setting to determine the natural course of pN1 disease following RP.

The aim of the present study is to evaluate the complications and oncological outcome of (s)e-PLND in combination with RP.

## Patients and methods

### Study population

Institutional review board approval was obtained with a waiver of informed consent. Between January 2016 and January 2020, 520 consecutive patients with clinically localized intermediate or high risk PCa scheduled for RARP underwent a concomitant (s)e-PLND. Clinical, procedural, and histopathological data were prospectively collected, i.e. initial serum PSA level, clinical T-stage determined by digital rectal exam, ISUP grade, number of (positive) biopsy cores. Patients were categorized according to the EAU risk classification [11].

To assess the risk of LNI, the 2018 MSKCC nomogram which included biopsy core information was employed retrospectively for the early cohort in our study when not documented, and prospectively in the later cohort. Notably, the MSKCC nomogram does not include data from MRI and target biopsies, so if multiple targeted biopsies were conducted on a single suspicious lesion, they were counted as a single (positive) biopsy core. Patients were included with an LNI risk of more than 5%. Patients with an unknown dissection template or those in whom fewer than ten LNs were removed were excluded. Additionally, individuals who had undergone an incomplete PLND (e.g., unilateral dissection due to previous surgery) were excluded, as were those who had undergone salvage PLND procedures following previous local prostate cancer treatment. Patient follow-up data were collected from electronic health records.

## Surgical procedure and histopathological evaluation

Four experienced surgeons [MS, EV, DS, JPvB] (each with ≥ 250 procedures experience at the start of the study) performed the procedures using the Da Vinci Xi (Intuitive Surgical, Sunnyvale, CA, USA) in a single-center. The e-PLND was defined as the bilateral removal of the fibrofatty lymphatic tissue within the obturator fossa, the internal iliac region, and overlying the external iliac artery and vein. The se-PLND was performed at the discretion of the surgeons and involved the additional removal of lymph nodes from the pre-sacral and/or common iliac regions. Additionally, the periprostatic fat was removed in all patients. Dissected lymph nodes were submitted separately per anatomical template for histopathologic examination. Pathological staging occurred as stated by the International Union for Cancer Control (IUCC) Tumor-Node-Metastasis (TNM) 8th classification system by two dedicated uropathologists. The total LN yield and number of LN metastases were recorded in relation to the anatomical locations.

## Outcomes

The primary outcome was the histopathological outcome of (s)e-PLND versus the risk of complications (≤ 90 days) classified using the Clavien-Dindo (CD) grading system [19]. Complications attributed to (s)e-PLND included symptomatic lymphocele, lymphedema, ureter damage, nerves, and iliac vessels. Secondary outcomes encompassed peri- and post-procedural factors, such as blood loss, operative time, length of hospital stay, number of resected LNs, and number of metastatic LN. Additionally, biochemical recurrence rates (BCR) were evaluated. BCR was defined as two consecutive PSA values of ≥ 0.2 ng/mL, and disease-free survival measured the time from RARP with (s)e-PLND until BCR or the date of the last follow-up. In patients with pN1 disease subsequent treatment decisions were based on serum PSA velocity, imaging results, and further discussed in a multidisciplinary tumor board meeting.

### Statistical analysis

Descriptive statistics included medians and interquartile ranges (IQR) for continuous variables and frequencies with percentages for categorical variables. Differences between e-PLND and se-PLND were evaluated using Fisher’s exact test for categorical variables and the Mann-Whitney U test for continuous variables. Cox regression analysis was employed to assess the relationship between pre- and postoperative characteristics and the risk of BCR. BCR-free survival was visualized using a Kaplan-Meier curve. All tests were two-tailed, with statistical significance set at *p* < 0.05. Data analysis was performed using SPSS software version 27 (SPSS Inc., Chicago, IL, USA).

## Results

### Patients and histopathological characteristics

A total of 354 eligible patients were included in the study (supplementary Fig. 1). Of these, 180 patients (51%) underwent an se-PLND. Table 1 provides detailed information on patient characteristics, biopsy histopathology, EAU risk stratification, and MSKCC LNI risk prediction. The median calculated MSKCC LNI risk was 18% (IQR of 9–31%).

**Table 1:** Clinical patient characteristics

Characteristics	Total	e-PLND	se-PLND	
Number of patients, n (%)	354 (100%)	174 (49%)	180 (51%)	
Median age at surgery, years (IQR)	66 (62–70)	66 (62–70)	63 (62–70)	*NS*
Median PSA level at diagnosis, ng/ml (IQR)	9.0 (6.5–14)	9.3 (6.4–15)	8.6 (6.6–12)	*NS*
Clinical T-stage, n (%) cT1c cT2 cT3	135 (38%) 174 (49%) 45 (13%)	69 (40%) 86 (49%) 19 (11%)	66 (37%) 88 (49%) 26 (14%)	*NS*
Biopsy ISUP grade group/Gleason score, n (%) ISUP grade group 1/ Gleason score 6 ISUP grade group 2/ Gleason score 3 + 4 = 7 ISUP grade group 3/ Gleason score 4 + 3 = 7 ISUP grade group 4/ Gleason score 8 ISUP grade group 5/ Gleason score 9–10	6 (1.7%) 90 (25%) 104 (29%) 80 (23%) 74 (21%)	5 (2.9%) 39 (22%) 53 (31%) 40 (23%) 37 (21%)	1 (0.56%) 51 (28%) 51 (28%) 40 (22%) 37 (21%)	*NS*
Median number of biopsies, (IQR)	12 (8–12)	12 (6–12)	12 (10–13)	0.003
Median number of positive biopsies, (IQR)	5 (3–8)	5 (3–6)	6 (4–9)	< 0.001
EAU risk group, n (IQR) Intermediate risk High risk	149 (42%) 205 (58%)	71 (41%) 103 (59%)	78 (43%) 102 (57%)	*NS*
Median MSKCC LNI risk, (IQR) MSKCC LNI risk ≥ 10%, n (%) MSKCC LNI risk ≥ 20%, n (%) MSKCC LNI risk ≥ 30%, n (%)	18 (9–31) 269 (74%) 160 (45%) 93 (26%)	18 (9–33) 128 (74%) 79 (45%) 48 (28%)	18 (10–29) 141 (77%) 81 (44%) 45 (25%)	*NS*
Pre-operative PSMA conducted, n (%) Suspected lymph nodes (cN1), n (% of PSMA conducted)	127 (36%) 27 (21%)	52 (33%) 10 (19%)	75 (46%) 17 (23%)	*NS* *NS*

*Percentages may not total 100 because of rounding. Mann–Whitney U-test was used for continuous variables. Fisher’s exact test was used for categorical variables

EAU = European Association of Urology; ISUP = International Society of Urological Pathology; IQR = Interquartile range; LNI = Lymph node invasion; MSKCC = Memorial Sloan Kettering Cancer Center; PSA = prostate-specific antigen; (s)e-PLND= (super) extended Pelvic Lymph Node Dissection

Table 2 presents histopathological, perioperative, and postoperative outcomes. In total, 8,174 LN (8,247 including periprostatic LN) were resected, with a median of 22 (IQR 16–28) LN removed per patient. In total, 77 patients (22%) had pN1 disease with a median of 2 (IQR 1–3) metastatic lymph nodes per patient with pN1. A combined total of 186 metastatic lymph nodes (197 including periprostatic lymph nodes) were excised. The distribution of lymph node metastases according to anatomical regions is illustrated in supplementary Fig. 2.

**Table 2:** Perioperative and histopathological outcomes

	Total (*n* = 354)	e-PLND (*n* = 174)	se-PLND (*n* = 180)	*p*-value
Median time of surgery, minutes (IQR)	190 (151–222)	180 (142–216)	208 (180–240)	< 0.001
Median blood loss, ml (IQR)	200 (150–300)	200 (100–300)	230 (150–350)	*NS*
Median hospital length of stay, days (IQR) length of stay ≥ 4 days, n (%)	2 (2–2) 32 (9%)	2 (2–2) 18 (10%)	2 (1–2) 14 (7.8%)	*NS*
Complications attributable to PLND, n (%) No Yes, any complication Yes, Clavien-Dindo Grade ≥ 2	271(77%) 83 (23%) 36 (10%)	141 (81%) 33 (19%) 19 (11%)	130 (72%) 50 (28%) 17 (9.4%)	0.060 *NS*
Clavien-Dindo classification (< 90 days), n (%) Grade I Grade II Grade IIIa Grade IIIb Grade IV Grade V	47 (13%) 9 (2.5%) 20 (5.6%) 3 (0.8%) 4 (1.1%) -	14 (8%) 3 (1.7%) 11 (6.3%) 2 (1.1%) 3 (1.7%) -	33 (18%) 6 (3.3%) 9 (5.0%) 1 (0.6%) 1 (0.6%) -	*NS*
RARP ISUP grade group/Gleason score, n (%) ISUP grade group 1/ Gleason score 6 ISUP grade group 2/ Gleason score 3 + 4 = 7 ISUP grade group 3/ Gleason score 4 + 3 = 7 ISUP grade group 4/ Gleason score 8 ISUP grade group 5/ Gleason score 9–10	8 (2.3%) 107 (30%) 139 (39%) 47 (13%) 53 (15%)	5 (2.9%) 50 (32%) 66 (41%) 27 (11%) 26 (15%)	3 (1.7%) 57 (32%) 73 (41%) 20 (11%) 27 (15%)	*NS*
RARP Tumor stage, n (%) pT2 pT3a pT3b pT4	116 (33%) 153 (43%) 80 (23%) 5 (1.4%)	66 (38%) 65 (37%) 40 (23%) 3 (1.73%)	50 (28%) 88 (49%) 40 (22%) 2 (1.1%)	*NS*
Positive surgical margins, n (%)	122 (34%)	65 (37%)	57 (32%)	*NS*
Median number of dissected lymph nodes, n (IQR)	22 (16–27)	17 (13–24)	25 (20–32)	< 0.001
Nodal stage, n (%) pN0 pN1	277 (78%) 77 (22%)	150 (86%) 24 (14%)	127 (71%) 53 (29%)	< 0.001
Median number of metastatic lymph nodes in case of LNI, n (IQR)	2 (1–2)	2 (1–2)	2 (1–3)	*NS*

*Percentages may not total 100 because of rounding. (Mann–Whitney U-test was used for continuous variables. Fisher’s exact test was used for categorical variables)

ISUP = International Society of Urological Pathology; IQR = Interquartile range; LNI = Lymph node invasion; PSA = prostate-specific antigen; RARP = Robot-assisted radical prostatectomy; (s)e-PLND= (super) extended Pelvic Lymph Node Dissection

The periprostatic fat contained lymph nodes in 17% of patients (61 out of 354). Among those with periprostatic LN, 18% (11 out of 61) had metastatic disease. In four patients (1.1% of the total study cohort) periprostatic lymph nodes represented the sole region of lymph node metastasis.

## Extended versus super-extended PLND

Of the total 197 metastatic LNs, 28 (14%) were situated outside the standard e-PLND template, however, in all-but-one patient (with one metastatic common iliac LN), there was also concomitant LNI in the e-PLND template (Fig. 2). pN1 disease was detected in 29% (53/180) of men who underwent se-PLND compared to 14% (24/174) of men undergoing e-PLND (*p <* 0.001). Tumor stage, Gleason Grade and margin status were equal between both groups. In both groups, the median number of positive LNs was two and did not differ significantly.

The median number of LNs resected was higher for se-PLND compared to e-PLND, 25 (IQR 20–32) versus 17 (IQR 13–24), respectively (*p* < 0.001) (Table 1). The predicted LNI risk according to the MSKCC nomogram was equal among the e-PLND and se-PLND cohorts, as were the other clinical characteristics, besides the median number of (positive) biopsies (Table 1).

Hospital length of stay and blood loss were equal among both groups. Complications of any grade were observed more often in patients who underwent se-PLND (19% vs. 28%), although not statistically significant (*p* 0.060). More severe complications (CD grade ≥ 2) were also not significant different between both groups. Lymphoedema was the most prevalent complication and lymphoceles the most prevalent complication requiring an intervention (supplementary Table 1). The median operation time of se-PLND was 28 min compared to e-PLND (208 vs. 108 min respectively; *p* < 0.001).

## Oncological follow-up

After excluding 47 men who were lost to follow-up, BCR rates were equal at 35% for both ePLND and se-PLND after a median follow-up of 24 months (IQR 7–33) (Supplementary Fig. 3). Upon multivariate analyses, clinical and biopsy characteristics were not prognostic of BCR. Pathological T-stage (pT2 versus pT3a, and pT2 versus pT3b), and the ISUP score of RARP were predictive of BCR (*p < 0.01*), whereas a positive surgical margin was not associated (*p* = 0.58). A total of 31 patients with pN1 disease (44%) were BCR free after 21 months of follow-up.

## Discussion

knowledge of the LNI status benefits patients in two distinct ways [20]. Firstly, it may aid in optimizing post-surgical management, guiding the extent of salvage radiotherapy (with or without pelvic irradiation) in case of disease recurrence. The question whether PLND offers any ‘direct’ therapeutic advantage in terms of improving progression free survival (PFS) remains a point of controversy. Our study demonstrates extending the PLND template to a super-extended dissection template improved the LN yield without increasing the rate of severe complications. Yet, it does lead to longer operative times, which brings associated costs.

A systematic review and meta-analysis conducted in 2017 did not find significant differences in oncological survival between men with ePLND and those without [14]. Two randomized controlled trials reported that e-PLND improved N-staging compared to limited-PLND but did not improve PFS using the extended template after a median follow-up of 3.1 years and 5 years respectively [17, 18]. Even though these trials compared a limited- or no PLND with an e-PLND template, their results align with our findings that extending the PLND template does not decrease the BCR risk at intermediate-term follow-up. Nevertheless, a substantial number of men with histologically proven LNI, as high as 44% in our cohort, did not develop BCR. This is consistent with the findings by Marra et al. who reported a BCR-free survival rate of 28–56% depending on the length of follow-up [21], suggesting a therapeutic benefit from the PLND.

While e-PLND would have correctly staged nearly all patients with LNI in our series, the inclusion of the presacral and common iliac regions in the template resulted in the removal of an additional 14% of metastatic LN. These findings align with prior research supporting the use of the se-PLND template in selected patients [15, 22].

The indication and the extent of PLND remains a challenge. An international, multicenter study incorporated PSMA-PET/CT into existing nomograms in order to predict LNI better than the nomograms recommend by the EAU guideline. Performance of the MSKCC nomogram and Briganti nomograms was assessed in 757 patients undergoing RARP and e-PLND. Addition of PSMA-PET/CT to the nomograms substantially improved the discriminative ability of the models [23]. However, despite an improved area under the receiver operating curve of the Briganti 2019 nomogram and the PSMA-incorporated model by Meijer et al., the clinical net benefit at a lower risk threshold remains limited [24, 25].

In the Netherlands, the prospective randomized trial PSMA-SELECT is currently conducted, based on the hypothesis that ePLND should only be performed in addition to RARP in case of LNI suspected on PSMA-PET/CT, ensuring this invasive intervention is reserved for men with suspected LNI. For those with negative PSMA-PET/CT, the possibility of the presence of small positive lymph nodes is accepted in this study. In cases of BCR during follow-up, a PSMA/PET-CT is performed and LNI can treated when visible. This approach suggests that initial ePLND may be safely omitted in men without LNI on PSMA, without compromising PCa specific survival [26]. To detect PSMA visible LNI during surgery, PSMA-guided robot-assisted PLND may be helpful, especially when suspected lymph nodes are located outside the standard ePLND template [27].

Strengths of our study include a substantial number of consecutive patients in a contemporary, homogenous cohort without neoadjuvant treatment. The procedures were conducted by experienced urologists in a single-center, ensuring consistency in (s)e-PLND templates and providing valuable insights into lymph node metastases’ topography. Our study reinforces the clinical significance of removing and evaluating periprostatic fat, and we recommend its removal during RARP [28]. The PLND and histopathological analysis were performed in a high-volume setting, indicated by the high number of resected LNs compared to other series. Our study contributes to a limited body of research examining the benefits and harms of se-PLND compared to e-PLND.

Nonetheless, our study has limitations. Its retrospective design introduces inherent confounding biases in the selection of patients who underwent e-PLND or se-PLND. The recent introduction of PSMA-PET/CT, with its high specificity, may refine the PLND template in patients with suspected LNI. The sensitivity of PSMA-PET/CT remains too limited to avoid a PLND solely based on a negative PSMA-PET/CT. In the mid-term, we did not see an effect of se-PLND on BCR-free survival, but a longer follow-up is needed to analyze the long-term effect. Despite these limitations, our study contributes to our understanding of the value of (s)e-PLND during RARP.

## Conclusion

Compared to the standard extended template, the super-extended PLND (se-PLND) increases the number of dissected lymph nodes and consequently the detection of metastases. In the present series, se-PLND did not increase postoperative morbidity following robot assisted radical prostatectomy. The contribution of se-PLND to BCR-free survival seems to be very limited at intermediate term follow-up.

## Electronic supplementary material

Below is the link to the electronic supplementary material.

Supplementary Material 1

## Data Availability

Bas Israel and Diederik Baas had full access to all the data in the study and take responsibility for the integrity of the data and the accuracy of the data analysis.
