# Supplementary material for: Evaluation of complications and biochemical recurrence rates after (super) extended lymph node dissection during radical prostatectomy
Source: World J Urol. 2024 Oct 30;42(1):605. doi: 10.1007/s00345-024-05321-6 (PMC11525387; doi:10.1007/s00345-024-05321-6)
Supplement: Supplementary file 1 — Supplementary Material 1 [file 345_2024_5321_MOESM1_ESM.docx]

**Evaluation of complications and recurrence rates after (super) extended lymph node dissection during radical prostatectomy**

Diederik J.H. Baas^1,2,3*^, Bas Israël ^2,^**^*^**, Joost M.S. de Baaij^1,3^, Henricus J.E.J. Vrijhof^3,4^, Robert J.Hoekstra^3,4^, Heidi Kusters-Vandevelde^5^, Diederik M. Somford^1,3^, Peter F.A. Mulders^2^, J.P. Michiel Sedelaar^2,3^, Jean-Paul A. van Basten^1,3^

^1^ Department of Urology, Canisius Wilhelmina Hospital, Nijmegen, The Netherlands

^2^ Department of Urology, Radboud university medical center, Nijmegen, The Netherlands

^3^ Prosper Prostate Cancer Clinics, Nijmegen/Eindhoven, The Netherlands

^4^ Department of Urology, Catharina Hospital, Eindhoven, The Netherlands

^5^ Department of Pathology, Canisius Wilhelmina Hospital, Nijmegen, The Netherlands

^*^ These authors contributed equally to this work.

Corresponding author:

Diederik J.H. Baas, MD

Prosper Prostate Cancer Clinics

Department of Urology, Canisius Wilhelmina Hospital

Weg door Jonkerbos 100, 6532 SZ, Nijmegen, The Netherlands.

Email: d.baas@cwz.nl

**Supplementary figures and tables**

**Supplementary figure 1: Flow diagram of the study design and participants**

LNI= Lymph node invasion; e-PLND= extended pelvic lymph node dissection; se-PLND= super-extended pelvic lymph node dissection; RARP= robot-assisted radical prostatectomy.

**
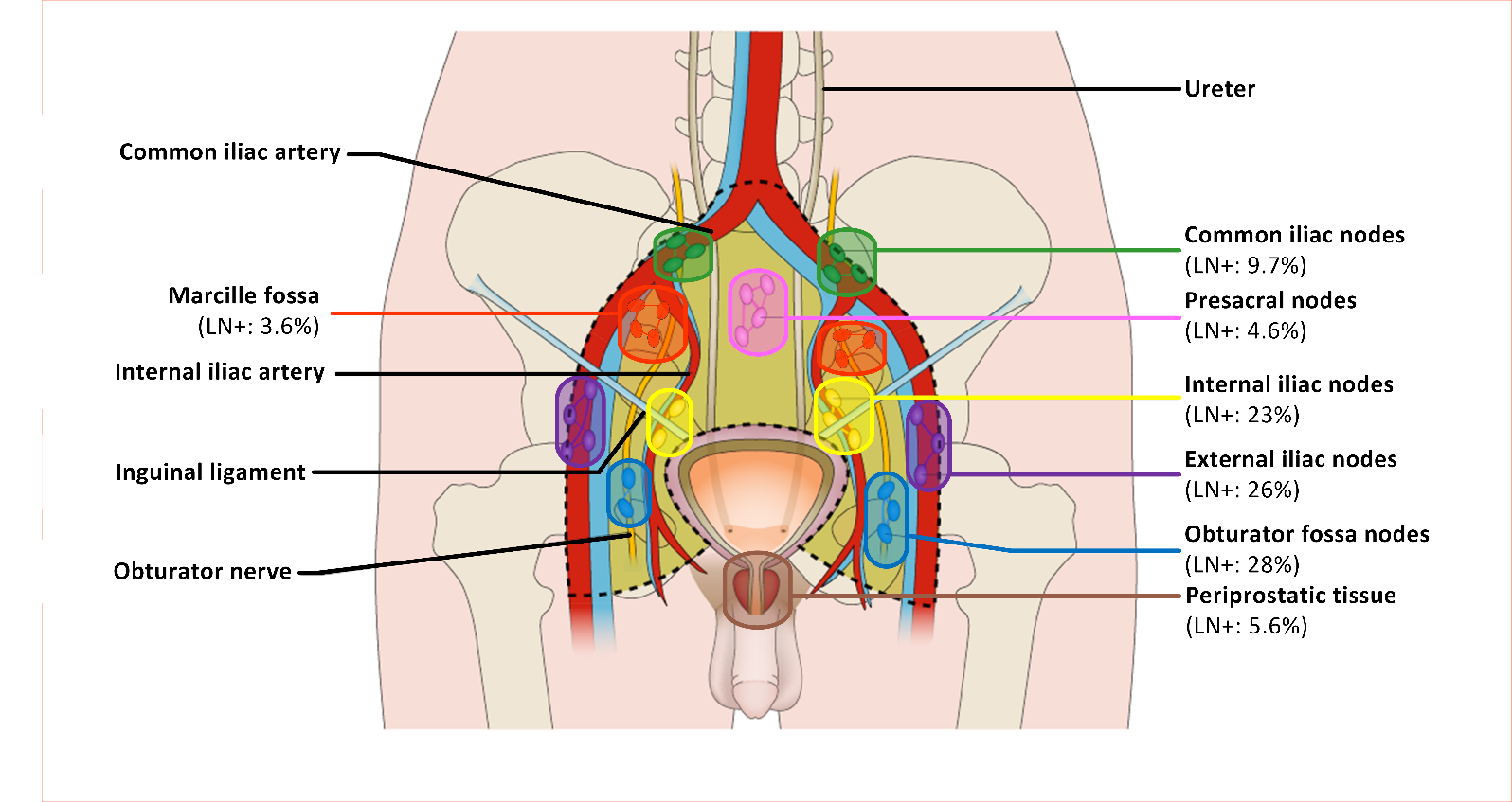
Supplementary figure 2: Lymph node metastases per anatomical region**

**Supplementary figure 3: Kaplan-Meier curve for biochemical recurrence free survival**

**
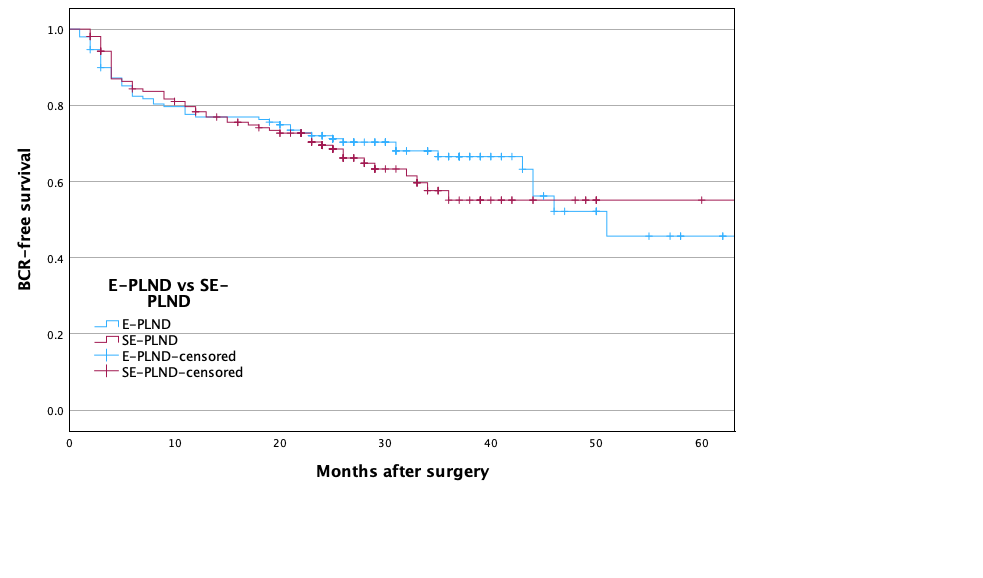
**

Blue curve represents e-PLND, the red curve shows the BCR free survival of se-PLND (Log-rank *p* 0.61)

BCR= biochemical recurrence; (s)e-PLND= (super) extended Pelvic Lymph Node Dissection.

**Supplementary table 1: overview of the complication types and Clavien-Dindo grading**

| **Complication** | **I** | **II** | **IIIa** | **IIIb** | **IV** | **Total** |
| --- | --- | --- | --- | --- | --- | --- |
| Lymphocele | 1 | 5 | 12 | - | - | 18 |
| Lymphoedema | 37 | 1 | - | 1 | - | 39 |
| Hematoma or bleeding | 4 | - | 2 | 1 | 2 | 9 |
| Ureter Injury | - | - | 3 | 1 | 1 | 5 |
| DVT/PE | - | 2 | 2 | - | 1 | 5 |
| Neuropraxia | 5 | 1 | 1 | - | - | 7 |
| Total | 47 | 9 | 20 | 3 | 4 | 83 |

Supplementary table 1: Overview of complication types and Clavien-Dindo grade group. DVT: deep venous thrombosis. PE: pulmonary embolism.

**Supplementary table 2: subsequent imaging in patients with biochemical recurrence (n=107)**

|  | **Total (n=107)** | **E-PLND (n=52)** | **SE-PLND (n=55)** |
| --- | --- | --- | --- |
| **Subsequent imaging, n (% of patients with BCR)** | 75 (71%) | 35 (67%) | 40 (73%) |
| PSMA | 67 | 33 | 34 |
| Bone scintigraphy | 4 | 1 | 3 |
| CT-scan | 5 | 2 | 3 |
| PET-scan | 4 | 2 | 2 |
| MRI scan | 4 | 1 | 3 |
| **Visible lesions** | 55 (73%) | 24 (69%) | 31 (78%) |
| Local recurrence | 16 | 8 | 8 |
| Locoregional lymph nodes | 43 | 18 | 25 |
| Distant | 13 | 10 | 3 |

Supplementary table 2: Subsequent imaging in patients with biochemical recurrence. PSMA: prostate-specific membrane antigen. PET: positron emission tomography. CT: computed tomography. MRI: magnetic resonance imaging. Of note: patients were able to receive multiple imaging modalities and able to have visible lesions on multiple locations. No significant differences between both groups were observed.

**Supplementary table 3: subsequent treatment in patients with biochemical recurrence**

**Supplementary table 3: subsequent treatment in patients with biochemical recurrence (n=107)**

|  | **Total (n=107)** | **E-PLND (n=52)** | **SE-PLND (n=55)** |
| --- | --- | --- | --- |
| **Subsequent treatment, n (% of patients with BCR)** | 57 (53%) | 27 (52%) | 30 (55%) |
| Radiotherapy | 45 | 22 | 23 |
| ADT | 36 | 20 | 16 |
| Chemotherapy | 1 | 0 | 1 |
| Salvage re-PLND | 3 | 0 | 3 |

Supplementary table 3: Subsequent treatment in patients with biochemical recurrence. ADT: androgen deprivation therapy. (S)E-PLND: (super) extended pelvic lymph node dissection. Of note: patients were able to receive multiple treatments combined, for example radiotherapy with ADT. No significant differences between both groups were observed.
